# A rapid and durable response to larotrectinib in a patient with *NTRK* fusion-positive secretory carcinoma originating from the external auditory canal

**DOI:** 10.1007/s13691-022-00559-6

**Published:** 2022-06-14

**Authors:** Yuichi Ando, Sachi Morita, Tomoya Shimokata, Toyonori Tsuzuki, Shigeru Inafuku, Kenichiro Iwami, Nicoletta Brega, Takashi Akagawa, Toshiaki Tsujino, Tetsuya Ogawa

**Affiliations:** 1 Department of Clinical Oncology and Chemotherapy, Nagoya University Hospital, 65 Tsurumai-cho, Showa-ku, Nagoya, 466-8550 Japan; 2 Department of Surgical Pathology, Aichi Medical University, 1-1 Yazakokarimata, Nagakute, Aichi 480-1195 Japan; 3 Department of Otorhinolaryngology-Head and Neck Surgery, Aichi Medical University, 1-1 Yazakokarimata, Nagakute, Aichi 480-1195 Japan; 4 Departments of Neurosurgery, Aichi Medical University, 1-1 Yazakokarimata, Nagakute, Aichi 480-1195 Japan; 5 Bayer Pharmaceuticals, Viale Certosa 130, 20156 Milan, Italy; 6 Bayer Yakuhin, Ltd., Breeze Tower, 2-4-9, Umeda, Kita-ku, Osaka, 530-0001 Japan

**Keywords:** *NTRK* fusion, Secretory carcinoma, Tropomyosin receptor kinase inhibitior, Larotrectinib, External auditory canal cancer

## Abstract

For decades, no clear consensus existed on the standard treatment option for malignant tumors of the external auditory canal, an extremely rare disease. Here we report the case of a 55-year-old female patient with secretory carcinoma that originated from the left external auditory canal. Magnetic resonance imaging (MRI) at baseline showed that the tumor had extended to the medulla oblongata despite surgical and radiation treatments for more than 20 years from the initial diagnosis. Based on the results of a next-generation sequencing test of a formalin-fixed paraffin-embedded surgical specimen indicating that the tumor harbored *ETV6–NTRK3* fusion, the patient was enrolled in a global basket study of larotrectinib, an oral selective tropomyosin receptor kinase (TRK) inhibitor. Three weeks after the start of larotrectinib treatment, MRI showed only small remnants of the tumor in the medulla oblongata and the patient’s headache before the treatment had disappeared. Subsequent MRI after 12 weeks of treatment confirmed the complete disappearance of the tumor. The patient repeated grade 2 flu-like symptoms related to treatment, but did not experience any other grade 2 or worse treatment-related adverse events. TRK inhibitors, such as larotrectinib, exert potent antitumor activity against neurotrophic tyrosine receptor kinase (*NTRK*) fusion-positive tumors in a tumor-agnostic manner. To the best of our knowledge, this is the first report on *NTRK* fusion-positive secretory carcinoma of the external auditory canal, and this report provides a valuable insight into the management of the extremely rare but now treatable malignancy.

## Introduction

Many patients with neurotrophic tyrosine receptor kinase (NTRK) fusion-positive tumors have considerably benefited from the advent of tropomyosin receptor kinase (TRK) inhibitors [1]. *NTRK* gene fusions have been identified as oncogenic drivers in a variety of tumors and the TRK inhibitors exert potent antitumor activity against the tumors in a tumor-agnostic manner. As the frequency of *NTRK* fusions is extremely rare in most tumors (< 1%), it is extremely crucial to establish when and how to identify patients with *NTRK* fusion-positive tumors so as to ensure that they benefit from the treatment opportunity with TRK inhibitors [2]. Conversely, certain rare tumors commonly harbor *NTRK* fusions, including infantile fibrosarcoma, cellular and mixed subtypes of congenital cellular mesoblastic nephroma, and secretory carcinomas of the breast and salivary glands. It is crucial to recognize which of these tumors are likely to harbor *NTRK* fusion and to triage patients who should be tested for *NTRK* fusion in the early stages of treatment. We are able to determine which tumors fall into this important category by accumulating firsthand evidence from each case report.

Hitherto, no clear consensus existed for decades on the standard treatment strategies for malignant tumors of the external auditory canal, an extremely rare disease [3, 4]. Patients with this disease have been empirically treated surgically with a risk of serious complications [5, 6]. In the era of cancer genomic medicine, a breakthrough treatment strategy is eagerly desired.

Here, we report the case of a patient with *NTRK* fusion-positive secretory carcinoma originating from the external auditory canal, who achieved a rapid and durable response to larotrectinib, an oral selective TRK inhibitor. This study was approved by the Institutional Ethics Committee (Approval No. 2021-0324) and a written informed consent of the patient was obtained for the publication of this manuscript.

## Case report

**Fig. 1:**
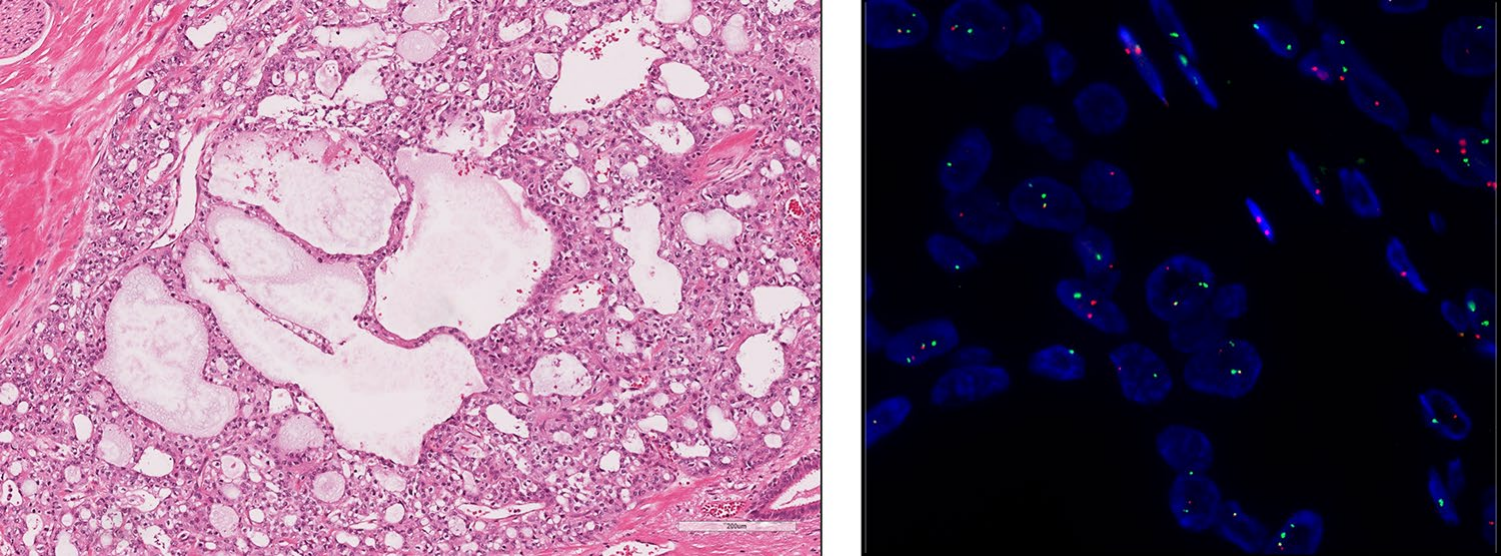
Histopathological examinations and fluorescence in situ hybridization (FISH) image of the surgical specimen. The tumor was well circumscribed but not encapsulated. No necrosis or hemorrhage was seen. The tumor showed a lobulated growth pattern separated by fibrous septa. It was associated with microcystic and tubular structural patterns with eosinophilic luminal secretions, typical findings of secretory carcinomas. The tumor cells were cuboidal with eosinophilic cytoplasm and round-to-oval nuclei (hematoxylin–eosin stain, left). FISH for *ETV6* showed split signals (red and green, right)

A 55-year-old female patient was referred to our institution as a potential candidate for an ongoing clinical study. The patient underwent surgery to remove a tumor originating from the left external auditory canal, diagnosed as a ceruminous adenocarcinoma 20 years before visiting our institution, followed by subtotal temporal bone resection, and postoperative radiation for intracranial recurrent tumor 12 years earlier, i.e., 8 years after the initial treatment. According to anecdotal medical records of the initial surgery, the histopathological diagnosis at that time was adenocarcinoma or acinic cell carcinoma. Five years before visiting our institution, i.e., 15 years after the initial treatment, the patient received stereotactic radiosurgery for the tumor that had extended to the petroclival region and the medulla oblongata. Moreover, 6 months before visiting our institution, the patient also underwent debulking surgery for recurrent tumors of the petroclival region and the medulla oblongata. Histopathological examinations of the surgical specimens from the medulla oblongata first detected adenocarcinoma that was consistent with the diagnosis of ceruminous adenocarcinoma and, moreover, that suggested secretory carcinoma (Fig. 1). The final histopathological diagnosis was secretory carcinoma taking into account the results of a split-signal indicating *ETV6* gene rearrangement on the fluorescence in situ hybridization (ZytoLight^®^, ZytoVision, Bremerhaven, Germany).

**Fig. 2:**
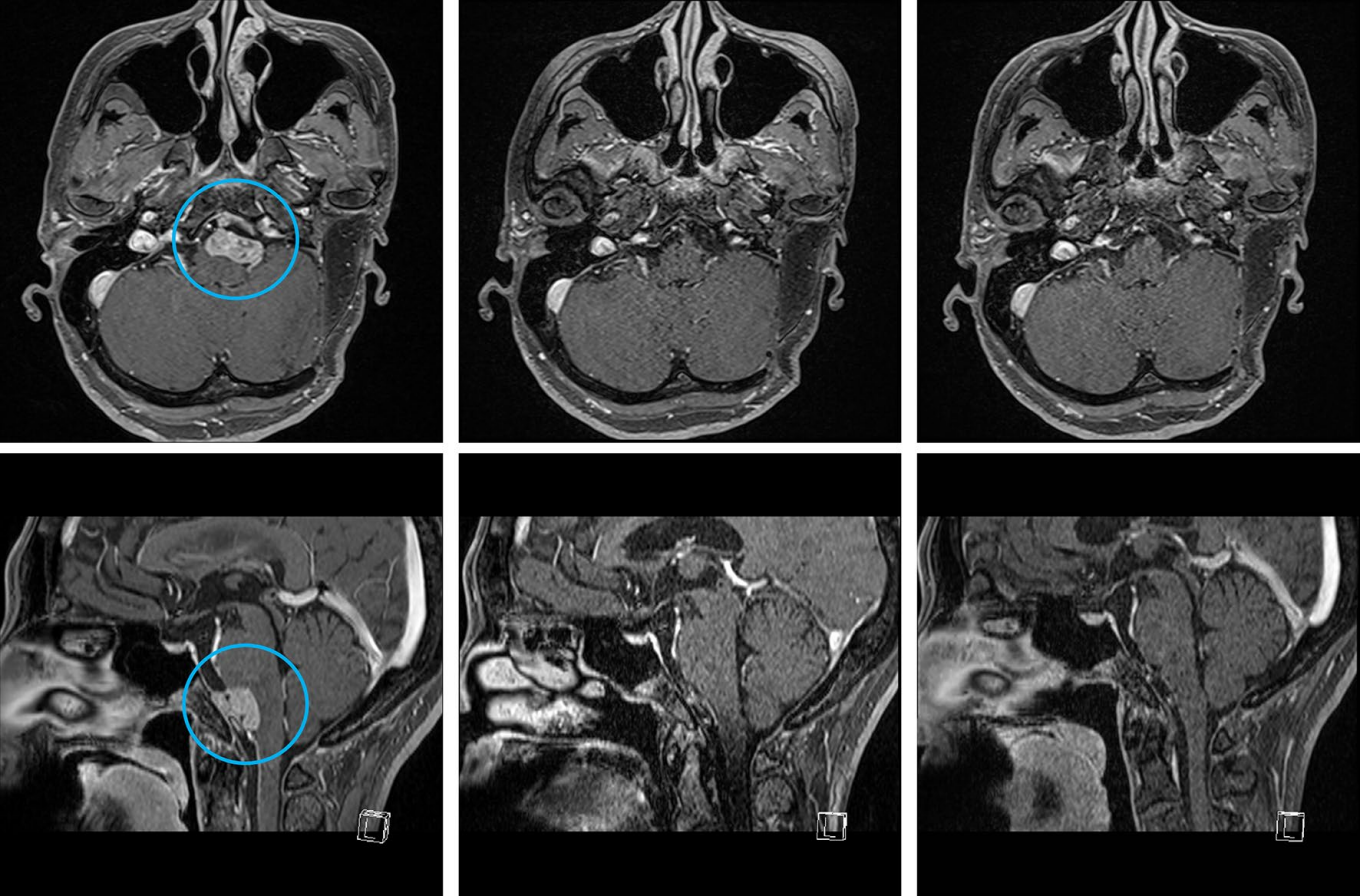
Gadolinium-enhanced T1-weighted magnetic resonance imaging (MRI). Axial (upper) and sagittal (low) MRI show that the tumor (circle) had extended to the left anterior of the medulla oblongata at baseline (left), remarkable regression at 3 weeks (middle), and complete disappearance at 12 weeks after the start of treatment with larotrectinib (right)

The patient was physically fine, had no abnormal laboratory findings, but experienced a mild headache on the left side that required regular use of an analgesic. Left-sided hearing loss, right-sided hearing impairment that required a hearing aid, and left facial paralysis were considered as complications of the previous surgeries. The patient was never a smoker and there was no family history suggestive of hereditary tumors. Magnetic resonance imaging (MRI) at baseline showed that the tumor had extended to the medulla oblongata (Fig. 2) and there was no clinical or radiographic evidence of distant metastases. The results of a comprehensive next-generation sequencing panel test (FoundationOne CDx^®^ Cancer Genomic Profile) of a formalin-fixed paraffin-embedded surgical specimen indicated that the tumor harbored *ETV6–NTRK3* fusion. Hence, the patient was enrolled in the NAVIGATE trial, a global basket study of larotrectinib; the study involved the administration of larotrectinib, an oral selective TRK inhibitor, at a dosage of 100 mg twice a day in a continuous 28-day cycle in adult patients with *NTRK* fusion-positive tumors (JapicCTI-194739, NCT02576431, Bayer Holding Ltd., Japan). Three weeks after the start of treatment with larotrectinib, MRI showed only small remnants of the tumor in the medulla oblongata (Fig. 2) and the patient’s headache had disappeared. A subsequent MRI after 12 weeks of treatment confirmed the complete disappearance of the tumor (Fig. 2). The patient repeated grade 2 flu-like symptoms related to treatment, but did not experience any other grade 2 or worse treatment-related adverse events according to the Common Terminology Criteria for Adverse Events (version 5.0). Other treatment-related adverse events, included grade 1 fatigue, nausea, anorexia, weight loss, increased alanine aminotransferase activity, and peripheral sensory neuropathy. At a follow-up visit after 19 months of treatment with larotrectinib, there was no evidence of tumor recurrence based on MRI. Hitherto, the patient has continued the treatment without any dose reduction or interruption and the adverse events are all well-controlled.

## Discussion

Larotrectinib exerts antitumor effects regardless of the primary sites of tumors provided that they harbor *NTRK* fusions. Hence, it is not surprising that the patient in this case report greatly benefited from the treatment. A recent pooled analysis of clinical studies on larotrectinib reported its long-term efficacy across a variety of cancer types. The objective response rate (ORR) was 75%, the median duration of response was 49.3 months, the median progression-free survival was 35.4 months, and the median time to response was 1.8 months [1]. The majority of adverse events observed in our patient were of grade 1, except the grade 2 flu-like symptoms that are easily manageable; this is also in line with the reported safety of larotrectinib [1]. Given the efficacy and safety profiles of larotrectinib, as well as entrectinib, patients with *NTRK* fusion-positive tumors should not miss the opportunities of receiving treatment with a TRK inhibitor. More importantly, this case corroborated the potent intracranial antitumor activity of larotrectinib, which resulted in a complete disappearance of the tumor in the medulla oblongata. Some case series reported a complete disappearance of central nervous system (CNS) metastases from a variety of cancer types due to treatment with larotrectinib. The referenced pooled analyses also showed that the ORR of CNS metastases was 73% which is comparable to that of extracranial lesions [1, 7]. The antitumor activity of lalotrectinib has also been reported in *NTRK* fusion-positive primary CNS tumors [8]. These findings provide ample evidence that patients with *NTRK* fusion-positive tumors should be treated with the systemic therapy before the local treatment for CNS metastases.

Malignant tumors originating from the external auditory canal account for less than 1% of all head and neck cancers, with an estimated global incidence of less than one per one million people annually. Of these, squamous cell carcinoma is the most common histopathological subtype and adenocarcinoma is even more rare [3, 4]. In recent case series of 22 and 27 patients with external auditory canal carcinoma, only one patient from each case had adenocarcinoma [5, 6]. According to old case series of 92 patients with primary tumors of the external and middle ear, five patients were diagnosed with low-or high-grade adenocarcinoma [9]. This extreme rarity hampered the development of a standard treatment and; therefore, patients with this disease have been empirically treated with surgical treatment at the risk of severe complications, especially postoperative wound dehiscence and infection, hearing loss, facial paralysis and disfigurement [5, 6]. Although chemoradiotherapy may be an alternative to surgical treatment, the quality of evidence to support it, especially with respect to long-term prognosis, is insufficient [10, 11]. On the other hand, the histopathological origins of glandular tumors of the external auditory canal, namely, ceruminous glands (modified apocrine glands located in the cartilaginous segment of the external auditory canal), eccrine glands, or ectopic salivary glands, have been controversial for decades [8, 12–14]. Secretory carcinoma of the salivary glands (formerly known as mammary analog secretory carcinoma) is a low-grade salivary carcinoma characterized by a specific *ETV6* rearrangement. The histopathological findings are similar to those of the breast tumor, e.g., microcystic and solid or tubular structures with unique secretions. This tumor is usually positively stained with S-100, mammaglobin, CK7, MUC4, and GATA3, while in our patient the final diagnosis was based on the presence of *ETV6* rearrangements which did not require immunohistochemical staining. Before the secretory carcinoma of the salivary glands was well-identified, the tumor was sometimes misdiagnosed as acinic cell carcinoma, mucoepidermoid carcinoma, adenocarcinoma, or not otherwise specified. In any case, like the secretory carcinomas of the breast and salivary glands, the secretory carcinomas of the external auditory canal may share characteristics that they commonly harbor *NTRK* fusions regardless of their primary sites. Therefore, what is important in the era of cancer genomic medicine is to recognize that a subtype with *NTRK* fusion exists among the very rare malignancies. With an accurate molecular diagnosis of *NTRK* fusion at the early stage of treatment, our patient would have received treatment with a TRK inhibitor to maintain her quality of life.

In conclusion, we report the first case of *NTRK* fusion-positive secretory carcinoma originating from the external auditory canal. The patient achieved a rapid and durable response to larotrectinib, representing a dramatic advancement of genomic medicine. This case provides a valuable insight into the management of this extremely rare but now treatable malignancy.

## References

[1] Hong DS, Shen L, van Tilburg CM (2021). Long-term efficacy and safety of larotrectinib in an integrated dataset of patients with TRK fusion cancer. J Clin Oncol.

[2] Solomon JP, Benayed R, Hechtman JF, Ladanyi M (2019). Identifying patients with *NTRK* fusion cancer. Ann Oncol.

[3] Moody SA, Hirsch BE, Myers EN (2000). Squamous cell carcinoma of the external auditory canal: an evaluation of a staging system. Am J Otol.

[4] Lobo D, Llorente JL, Suárez C (2008). Squamous cell carcinoma of the external auditory canal. Skull Base.

[5] Park JM, Kong JS, Chang KH (2018). The clinical characteristics and surgical outcomes of carcinoma of the external auditory canal: a multicenter study. J Int Adv Otol.

[6] Correia-Rodrigues P, Ramalho S, Montalvão P, Magalhães M (2020). External auditory canal carcinoma: clinical characteristics and long-term treatment outcomes. Eur Arch Otorhinolaryngol.

[7] Rosen EY, Schram AM, Young RJ (2019). Larotrectinib demonstrates CNS efficacy in TRK fusion-positive solid tumors. JCO Precis Oncol.

[8] Doz F, van Tilburg CM, Geoerger B (2021). Efficacy and safety of larotrectinib in TRK fusion-positive primary central nervous system tumors. Neuro Oncol.

[9] Dehner LP, Chen KT (1980). Primary tumors of the external and middle ear Benign and malignant glandular neoplasms. Arch Otolaryngol.

[10] Shiga K, Nibu KI, Fujimoto Y (2021). Sites of invasion of cancer of the external auditory canal predicting oncologic outcomes. Head Neck.

[11] Takenaka Y, Cho H, Nakahara S, Yamamoto Y, Yasui T, Inohara H (2015). Chemoradiation therapy for squamous cell carcinoma of the external auditory canal: a meta-analysis. Head Neck.

[12] Wetli CV, Pardo V, Millard M, Gerston K (1972). Tumors of ceruminous glands. Cancer.

[13] Markou K, Karasmanis I, Vlachtsis K, Petridis D, Nikolaou A, Vital V (2008). Primary pleomorphic adenoma of the external ear canal Report of a case and literature review. Am J Otolaryngol.

[14] Shinomiya H, Uehara N, Fujita T (2021). Phase I trial of concurrent chemoradiotherapy with docetaxel, cisplatin and 5-fluorouracil (TPF-CRT) for locally advanced squamous cell carcinoma of the external auditory canal. Eur Arch Otorhinolaryngol.

